# Resting-State Subjective Experience and EEG Biomarkers Are Associated with Sleep-Onset Latency

**DOI:** 10.3389/fpsyg.2016.00492

**Published:** 2016-04-12

**Authors:** B. Alexander Diaz, Richard Hardstone, Huibert D. Mansvelder, Eus J. W. Van Someren, Klaus Linkenkaer-Hansen

**Affiliations:** ^1^Department of Integrative Neurophysiology, Center for Neurogenomics and Cognitive Research, Vrije Universiteit AmsterdamAmsterdam, Netherlands; ^2^Neuroscience Campus AmsterdamAmsterdam, Netherlands; ^3^Department of Sleep and Cognition, Netherlands Institute for NeuroscienceAmsterdam, Netherlands; ^4^Department of Psychiatry, VU University Medical Center/GGZ inGeestAmsterdam, Netherlands

**Keywords:** Amsterdam Resting-State Questionnaire (ARSQ), consciousness, mind wandering, sleep, multilevel modeling

## Abstract

Difficulties initiating sleep are common in several disorders, including insomnia and attention deficit hyperactivity disorder. These disorders are prevalent, bearing significant societal and financial costs which require the consideration of new treatment strategies and a better understanding of the physiological and cognitive processes surrounding the time of preparing for sleep or falling asleep. Here, we search for neuro-cognitive associations in the resting state and examine their relevance for predicting sleep-onset latency using multi-level mixed models. Multiple EEG recordings were obtained from healthy male participants (*N* = 13) during a series of 5 min eyes-closed resting-state trials (in total, *n* = 223) followed by a period–varying in length up to 30 min–that either allowed subjects to transition into sleep (“sleep trials,” *n*_*sleep*_ = 144) or was ended while they were still awake (“wake trials,” *n*_*wake*_ = 79). After both eyes-closed rest, sleep and wake trials, subjective experience was assessed using the Amsterdam Resting-State Questionnaire (ARSQ). Our data revealed multiple associations between eyes-closed rest alpha and theta oscillations and ARSQ-dimensions *Discontinuity of Mind, Self*, *Theory of Mind, Planning*, and *Sleepiness*. The sleep trials showed that the transition toward the first sleep stage exclusively affected subjective experiences related to *Theory of Mind, Planning*, and *Sleepiness*. Importantly, sleep-onset latency was negatively associated both with eyes-closed rest ratings on the ARSQ dimension of *Sleepiness* and with the long-range temporal correlations of parietal theta oscillations derived by detrended fluctuation analysis (DFA). These results could be relevant to the development of personalized tools that help evaluate the success of falling asleep based on measures of resting-state cognition and EEG biomarkers.

## Introduction

Insomnia, the reduced ability to initiate or maintain sleep, is the most commonly occurring sleep disorder, with up to a third of the general populace experiencing at least a mild form and 6–10% even meeting diagnostic criteria for insomnia syndrome (Morin et al., 1994; Spiegelhalder et al., 2012; Baglioni et al., 2014). It, therefore, poses a costly societal problem, both in terms of psychophysical well-being as well as financial losses, e.g., treatment costs and diminished productivity (Daley et al., 2009, 2015). Although, pharmacological intervention remains common the associated side-effects along with a focus on combating symptoms rather than causes leave ample room for alternative strategies, such as psychological intervention (Morin et al., 1994; Murtagh and Greenwood, 1995) or manipulation of body temperature (Raymann and Van Someren, 2008).

Given the well-defined electrophysiology of sleep-onset and the transition toward deeper sleep stages and in light of recent technological advances, novel approaches to combating insomnia involving neurofeedback may be on the verge of becoming feasible practical solutions (Diaz et al., 2012). Besides physiological characteristics, subjective experience exhibits marked changes during the transition from wakefulness to sleep (Yang et al., 2010) and may play a significant role in determining the success of falling asleep (Harvey, 2002; Harvey and Greenall, 2003). Identifying contributing factors, both in terms of cognition and electrophysiology, associated with sleep-onset latency therefore could be of great value for the development of novel treatments based on neurofeedback.

A useful context for investigating these potential contributors is the frequently employed eyes-closed resting state, as it resembles the initial mental and physical condition of the waking state prior to sleep onset. The past decade has witnessed a tremendous interest in the study of resting-state neurophysiology (Linkenkaer-Hansen et al., 2005; Stam et al., 2006; Montez et al., 2009; Massar et al., 2014) and cognition (Smallwood and Schooler, 2006; Delamillieure et al., 2010; Killingsworth and Gilbert, 2010; McVay and Kane, 2010), largely sparked by the discovery that the resting-state is likely associated with a default-mode of brain functioning (Raichle et al., 2001; Raichle, 2006; Raichle and Snyder, 2007; Zhang and Raichle, 2010). Therefore, the principal motivation behind this study was to explore whether a combination of resting-state cognition and EEG measures can explain variability in sleep-onset latency, i.e., the time it takes for an individual to enter stage 1 sleep (Rechtschaffen and Kales, 1968). To address this question, we focused on alpha (8–12 Hz) and theta-band (4–7 Hz) EEG activity, given their well-studied association with cognition and pronounced reactivity to arousal changes (Başar et al., 2001; Grunwald et al., 2002; Hermens et al., 2005; Sauseng et al., 2010; Yang et al., 2010; Spiegelhalder et al., 2012; Mathes et al., 2014). To characterize resting-state cognition, we used the Amsterdam Resting-State Questionnaire (Diaz et al., 2013, 2014), which is a self-report survey developed to characterize thoughts and feelings in the resting state along 10 dimensions.

Our main research question is whether or not the resting-state provides both electrophysiological as well as cognitive information relevant to the prediction of sleep-onset latency (SOL). We begin by exploring the relationship between spectral and dynamical-systems biomarkers, such as the DFA-exponent, of theta and alpha-band activity (Linkenkaer-Hansen et al., 2005; Hardstone et al., 2012; Smit et al., 2013) and ARSQ dimensions (Diaz et al., 2013, 2014) during 5 min of eyes-closed rest (ECR). Subsequently, it was tested whether subjective experience after each sleep or wake trial could be explained from predictors obtained from these sleep or wake trials as well as the preceding ECR trial. In order to differentiate between effects of merely being at rest for extended periods of time and genuine sleep-onset effects, sleep trials were matched by trials of equal duration where the subject had not fallen asleep (wake trials). Finally, by combining the results of the previous two stages of analysis, we show how sleep-onset latency can be associated with cognitive and electrophysiological measures obtained from the preceding eyes-closed rest trial alone.

## Materials and methods

### Participants

Healthy male subjects (*n* = 13) were drafted from the VU University Amsterdam student population (Mage = 24 ± 4 years, range 18–32 years). Exclusion criteria were a history of neurological pathology, medication, drug/alcohol abuse, or scores >5 on the Pittsburgh Sleep Quality Index (Buysse et al., 1989). The study was approved by the Medical Ethical Assessment Committee (METc) of the VU Medical Center.

### Experimental design

**Figure 1:**
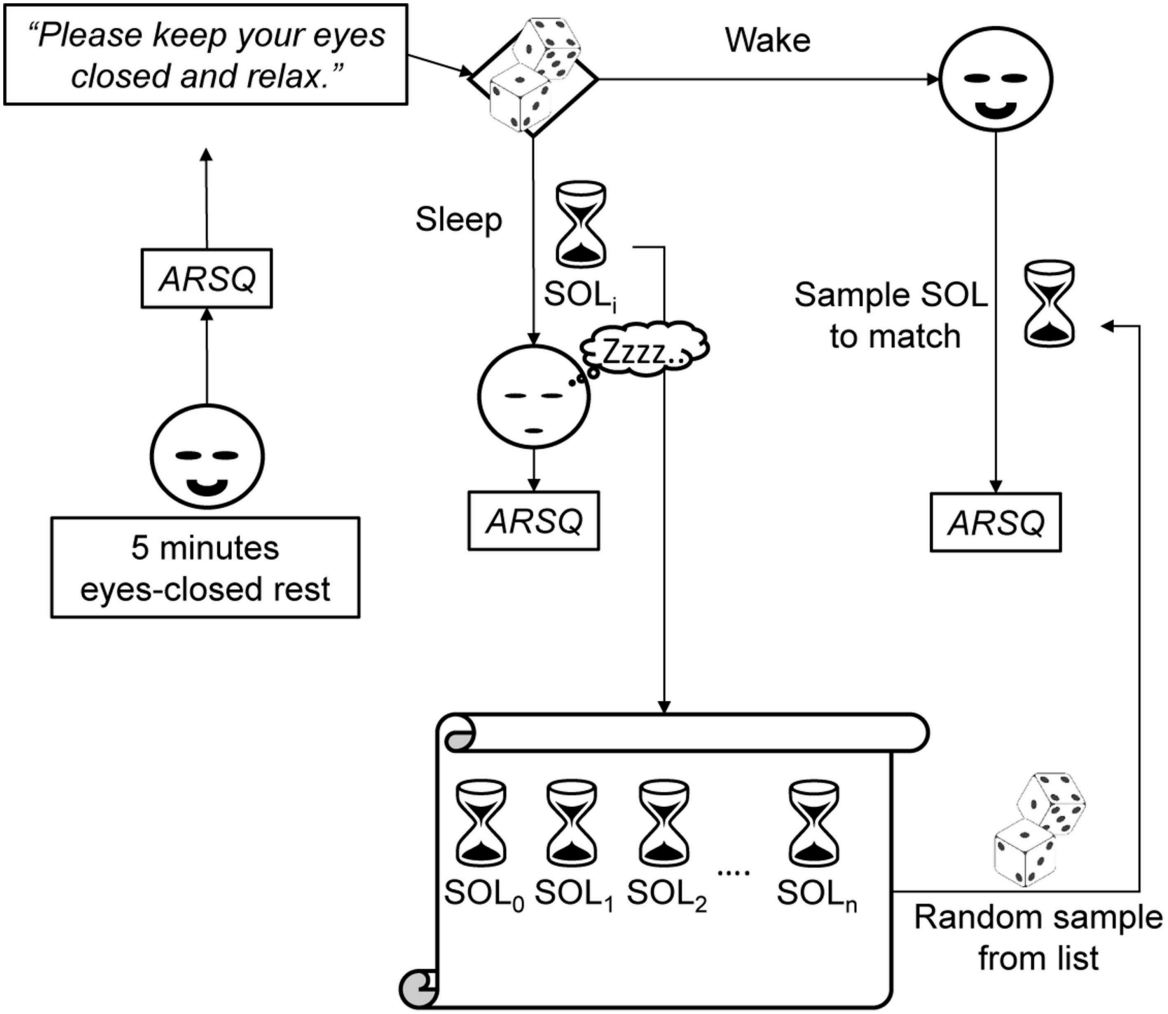
**Each experimental block consisted of a 5 min eyes-closed rest trial, and a subsequent sleep or wake trial (chosen at random) followed by the ARSQ**. Participants did not know beforehand whether they would be allowed to fall asleep. Therefore, in order to create a matching wake trial, a sleep-onset latency (SOL) was randomly selected from a list of previously obtained latencies and the awake participant would be interrupted after the selected time had passed. This matching would allow for differentiating between cognitive effects genuine to sleep-onset and those associated with merely spending time at rest.

Participants were scheduled for two visits to the EEG laboratory at the Center for Neurogenomics and Cognitive Research, each exactly 1 week apart and each starting between 9:00–10:00 A.M. On the first day, participants reviewed the instructions together with the experimenter and gave written informed consent prior to partaking in any of the experimental trials. During each visit, following EEG preparation, participants underwent up to nine experimental blocks that all started with a 5 min eyes-closed rest (ECR) trial during which subjects received the instruction “Please keep your eyes closed, relax, and try not to fall asleep.” Subsequently, subjects either participated in a sleep or a wake trial (Figure 1). In both cases, participants were lying in a comfortable bed in a dark room (20°C ambient temperature) for a maximum of 30 min. Although, informed about the various trial types, participants did not know which type of trial to expect, either sleep or wake, after the eyes-closed rest trial. Therefore, the only instruction given to participants was “Please keep your eyes closed and relax.”

In order to match the duration of sleep trials as best as possible, given that no explicit instruction was given to stay awake, a special procedure was followed (Figure 1). Before each experimental block, it was randomly determined whether the trial should be a sleep or wake trial. In case of a sleep trial, participants were allowed to fall asleep. In case of a wake trial, a sleep-onset latency was randomly selected from a list of previous, individual sleep-onset latencies. After the selected amount of time had passed, the participant was interrupted and asked to fill out the ARSQ and the matched SOL was removed from the list. This procedure necessitated that the first trial was a sleep trial (or otherwise no matching SOL could be derived for a subsequent wake trial, in which case the trial was discarded and retried). In addition, participants could fall asleep before the allotted time, in which case the trial counted as a sleep trial. In practice, this procedure resulted in a wake to sleep trial ratio of ~1:2, as participants tended to habituate to the experimental conditions and fell asleep more readily as time progressed (**Figure 3** and Table S2). In between experimental blocks participants were allowed rest and (self-selected) refreshments for 5–10 min. A 30 min break was offered halfway through the measurements, during which the EEG equipment was checked (e.g., electrode impedance). A successful sleep trial was defined as two consecutive 30 s periods of <50% alpha activity corresponding to 1 min of stage one (S1) sleep (Rechtschaffen and Kales, 1968) within a 30 min interval. The transition to S1 sleep was chosen because most individuals have been shown to still possess consciousness at this stage or are even unware they fell asleep (Yang et al., 2010). Immediately after each of the ECR and sleep/wake trials, subjects were asked to fill out the Amsterdam Resting-State Questionnaire (Diaz et al., 2013, 2014). The final data set contained a total of 446 observations (144 sleep trials and 79 wake trials and 223 preceding ECR trials) from 13 subjects over 2 measurement days.

### Assessment of resting-state cognition

Resting-state cognition was assessed using the ARSQ version 1.0 (Diaz et al., 2013), consisting of 50 items related to thoughts and feelings that may be experienced during (typically) a state of rest. All items were scored on a scale from “Completely Disagree” to “Completely Agree” on a 5-point scale. The ARSQ allows the derivation of the following cognitive dimensions by averaging the raw score over all corresponding items: Discontinuity of Mind, Theory of Mind, Self, Planning, Sleepiness, Comfort, Somatic Awareness, and Health Concern. All dimensions were obtained using the updated structure from the more recent ARSQ 2.0 (Diaz et al., 2014). The dimensions “Verbal Thought” and “Visual Thought” were not included in the analyses, as the complementary items introduced in the ARSQ 2.0 were not present in the current data set.

### Electrophysiology

**Figure 2:**
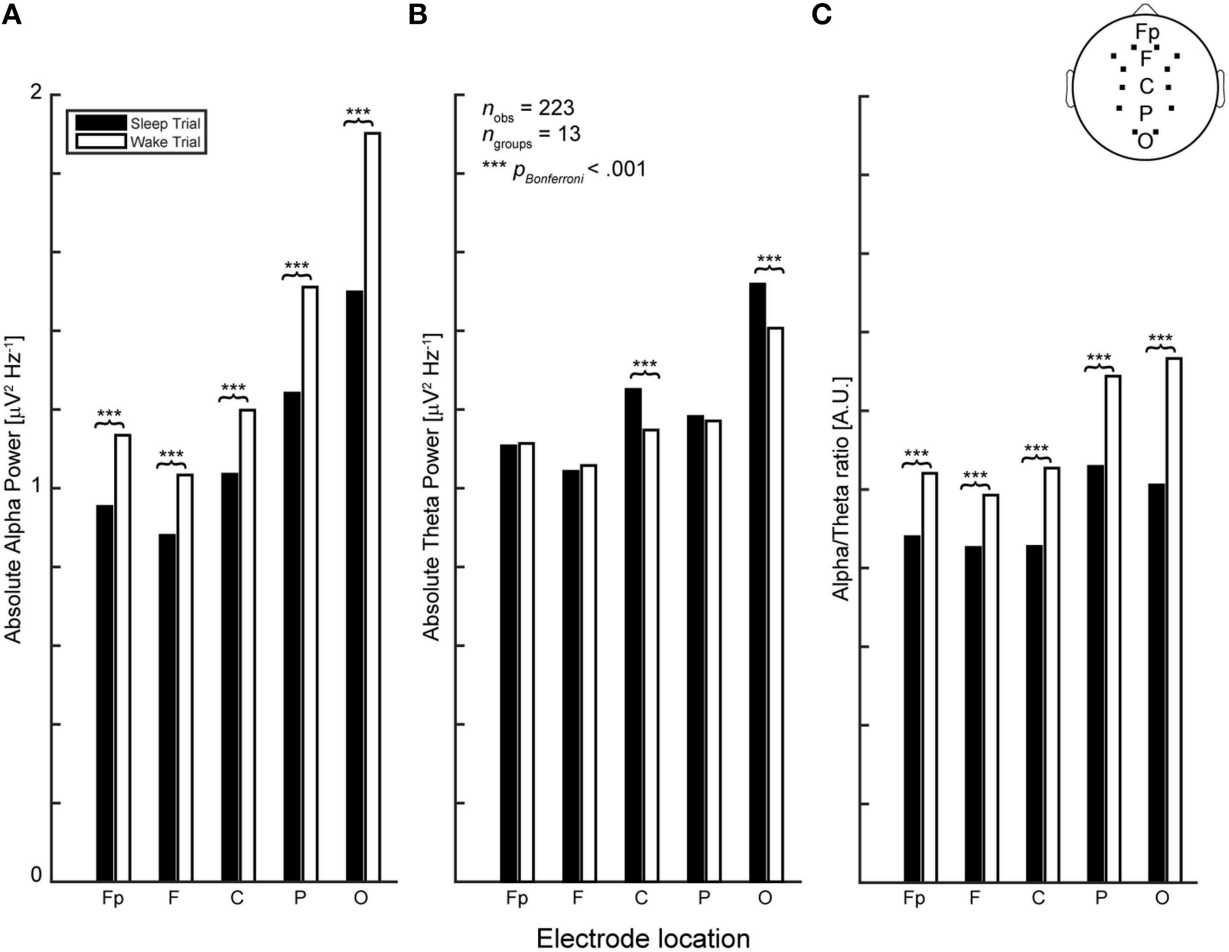
**In line with previous reports (Rechtschaffen and Kales, 1968; Hori et al., 1994) (A) EEG biomarkers of absolute band power in the alpha and (B) theta range exhibit an opposing relationship during sleep-onset at central and occipital electrode locations. (C)** To capture this quality in a single variable we included the alpha-theta ratio as additional biomarker variable.

High-density electroencephalographic recordings (EEG) were obtained using a 256 channel gel-based LTM HydroCel Geodesic Sensor Net coupled to a Net Amps 300 amplifier (Electrical Geodesics Inc., Eugene OR, USA). After EEG acquisition, EEG data were exported to MATLAB (The Mathworks Inc., Natick, MA), down-sampled from 1000 to 500 Hz and band-pass filtered between 1 and 45 Hz using finite impulse response (fir) filters. Data cleaning and artifact rejection was done using the automated algorithms in the MATLAB toolboxes FASTER (Nolan et al., 2010) to detect and interpolate bad channels and epochs and ADJUST (Mognon et al., 2011) to remove eye movement and muscle artifacts using independent component analysis. Signals were then re-referenced to common average reference. Analyses and EEG biomarker extraction was performed using EEGLAB (Delorme and Makeig, 2004) and the Neurophysiological Biomarker Toolbox (Hardstone et al., 2012). Here, we focused on theta-band (4–7 Hz) and alpha-band (8–12 Hz) absolute power and long-range temporal correlations as captured by detrended fluctuation analysis (DFA), over the complete duration of each recording (e.g., 5 min during eyes-closed rest and up to 30 min during sleep or wake trials) given their relevance in relating cognition to electrophysiology and sensitivity to arousal changes such as the transition into sleep (Rechtschaffen and Kales, 1968; Başar et al., 2001; Grunwald et al., 2002; Hermens et al., 2005; Linkenkaer-Hansen et al., 2005; Spiegelhalder et al., 2012; Feige et al., 2013). Furthermore, we included the alpha-theta ratio (absolute alpha-power/theta-power) as an additional biomarker, which we expected to be informative considering the inverse relationship between alpha and theta as sleep-onset approaches, i.e., increased theta power accompanied by alpha drop-out. This expectation was confirmed by testing the effect of trial condition (sleep or wake) on each (absolute power) biomarker (see Figure 2).

In order to restrict (model) complexity, the number of biomarker EEG-locations was reduced by first selecting only those channels corresponding to the 10–20 standard, except for temporal locations (as participants tended to sleep on their sides, resulting in strong muscle artifacts in the affected regions) and, subsequently, averaging the values of the 3 channels corresponding to frontal (F), parietal (P), central (C), occipital (O) regions, and 2 channels for the pre-frontal (Fp) region, because the pre-frontal electrodes are lateralized and contain only two channels in the 10–20 system. This resulted in five regions of averaged EEG power for the four selected biomarkers (Figure 2).

### Data analyses and statistics

The experimental design of this study was characterized by a large number of observations unequally distributed over the sleep and wake trials (*n* = 223, 144 sleep trials) as well as missing observations (partial drop-out of one participant) within a comparatively small sample of participants (*N* = 13). This prevents the use of classical regression and repeated-measures analysis of variance^1^ as the observations cannot be considered independent (Aarts et al., 2014). We therefore made use of (multi-level) linear mixed models (LMM) where residual variation does not necessarily exhibit independence and/or constant variance (Verbeke and Molenberghs, 2000; Xu, 2003; Jaeger, 2008; Quené and van den Bergh, 2008; Bolker et al., 2009; Chen et al., 2013). This is achieved by modeling random variation at different levels. Applied to this study, all trials (level 1) can be grouped within subjects (level 2). Conceptually, one may imagine a separate regression line being fit through all the trials of each subject, i.e., providing individual slope and intercept terms. These individual slope and intercept terms may be viewed as random normal fluctuations around an estimated overall mean (see Figure S1) and are hence referred to as random effects. Other explanatory variables, such as subject age, ARSQ-rating or EEG biomarker values, are treated similar to the predictors in classical regression and are denoted fixed effects. Fixed effect variation was furthermore explicitly partitioned into within- and between subject variation (van de Pol and Wright, 2009) using within-group centering. This made it possible to attribute effects to within-subject variability or between-subject differences (Figure S2).

**Figure 3:**
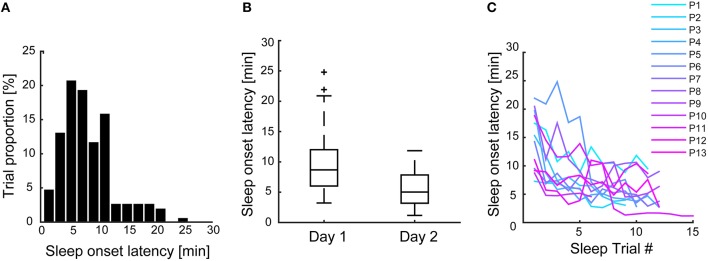
**Pooling all sleep trials shows that participants tended to fall asleep within the first 10 min of the recording (A), with an average sleep onset latency of 8 min**. However, sleep onset latency shortened over experimental days **(B)** and over subsequent sleep trials **(C)**, suggesting significant (individual) habituation over the course of the experiment.

We specified a separate multi-level model for each of the eight ARSQ-dimensions, with random effects being the subject id (intercept) and the experimental day (slope). As we expected habituation to occur over days (Figures 3B,C, this specification allowed for individual variation in the rate of habituation (i.e., the slope coefficient) and its correlation to the average response (e.g., allowing for high ratings to correlate with high increases over time). Model fit was assessed by a χ^2^-difference test, i.e., only those predictors were retained in the model that resulted in a significant increase in fit compared to a reduced version of the model, with the base model only including the intercept and random effect of subject id and experimental day.

Data preparation and analysis were performed in both MATLAB 2014a (The Mathworks Inc., Natick, MA) and R 3.1.1. (R Core Team, 2014). Testing the relationship between ARSQ rating, EEG biomarkers and sleep-onset latency was performed in R, primarily using the “lme4” package (Bates et al., 2014) for model estimation supported by the “pbkrtest” package (Halekoh and Højsgaard, 2014) for deriving degrees of freedom and *p*-values based on Kenward-Roger approximation. Finally, centered subject age, experimental day, trial duration (equivalent to sleep-onset latency for sleep trials, in minutes) and overall experiment duration on a given day (in hours starting from the first ECR recording) were included as covariates.

## Results

### Resting-state EEG biomarkers are associated with subjective experience

In order to assess the effect of resting-state brain activity on subsequent ARSQ-ratings, we fit a separate linear mixed model (LMM) for each cognitive dimension, in which its post-eyes-closed rest (ECR) ARSQ-rating was regressed on EEG biomarkers (separated into within-, and between-subject components) and potential confounders, i.e., experimental day, experiment duration on a given day, and participant age (see Materials and Methods). The general form of these models (where subscript “i” denotes the ARSQ-dimensions 1 to 8) was:

$$\begin{array}{l} {\text{ARSQ}}^{\text{ECR}}{}_{\text{i}}={\text{EEG}}_{\text{within}}+{\text{EEG}}_{\text{between}}+\text{Age}+\text{Day}+\\ \text{ Duration}+\text{random effects} \end{array}$$

We found (see Table 1) significant associations between EEG biomarkers and six of the eight dimensions from the ARSQ. Both the occipital theta-band DFA exponent (positive) and parietal alpha/theta-ratio (negative) were significant within-subject predictors of post eyes-closed rest ratings of Discontinuity of Mind. Alpha-power appeared a significant positive within-subject predictor for Theory of Mind (frontal) and Comfort (parietal). Frontal theta-power was a significant positive within-subject predictor for Self, whereas parietal theta-power significantly predicted Sleepiness, also restricted to the within-subject effect. Finally, the alpha/theta-ratio exhibited significant positive within-subject and between-subject effects on Sleepiness, but interestingly and opposing within-subject (positive) and between-subject (negative) effect on Planning. Apparently an increased alpha-theta ratio may effect increased ratings on Planning across the observations of a single participant, whereas between participants a higher average ratio is associated with less planning.

**Table 1:** **Resting-state brain activity in the alpha and theta bands offers significant predictors of post eyes-closed rest ratings on six ARSQ-dimensions**.

**Post-ECR ARSQ-rating: (*n* = 223)**	**Discontinuity of Mind**	**Theory of Mind**	**Self**	**Planning**	**Sleepiness**	**Comfort**
Degrees of freedom^a^	21	22	16	18	33	15
**FIXED EFFECTS (COEF.EST**. ± **SE)**						
Participant age	0.06±0.02^*^					
**DAY**						
Duration [hours]					−0.15 ± 0.03^***^	
**Theta abs. power^b^**			**Frontal**		**Parietal**	
Within-subject effect			0.55 ± 0.24^*^		1.02 ± 0.34^**^	
Between-subject effect			0.06±.42		0.19±.23	
**Theta DFA-exponent**	**Occipital**					
Within-subject effect	3.17 ± 1.22^*^					
Between-subject effect	3.72 ± 3.41					
**Alpha abs. power**		**Frontal**				**Parietal**
Within-subject effect		1.09 ± 0.35^**^				0.39 ± 0.11^***^
Between-subject effect		−0.14 ± 0.29				−0.10 ± 0.14
**Alpha/Theta ratio**	**Parietal**			**Parietal**	**Parietal**	
Within-subject effect	−0.54 ± 0.19^**^			0.76 ± 0.28^**^	−0.96 ± 0.21^***^	
Between-subject effect	−0.20 ± 0.20			−0.62 ± 0.24^*^	−0.77 ± 0.33^*^	
**RANDOM EFFECTS (SD)**						
Participant ID (β_0_)	0.25	0.55	0.47	0.52	0.63	0.48
Day (β_1_)	0.24	0.58	0.16	0.62	0.26	0.07
Correlation β_0_,β_1_	0.03	−0.56	−0.39	−0.89	−0.34	−0.06
Residual	0.56	0.76	0.60	0.89	0.70	0.43
Explained variance ${\left(\Omega_{0}^{2}\right)}^{}$^c^	0.41	0.42	0.47	0.35	0.54	0.61

*NB, Shown are estimated model coefficients ± Standard Error (the fixed effects, excluding intercept term) and random effect estimates*.

a *Degrees of freedom and p-values based on Kenward-Roger approximation*.

b *EEG Biomarkers were within-group centered (van de Pol and Wright, 2009)*.

c *Approximates oveHall model fit, similar to R^2^ in classical regression (Xu, 2003)*.

* *p ≤ 05*,

** *p < 0.01*,

*** *p < 0.001*.

*Significant results in bold face*.

### Sleep-onset reduces theory of mind and planning

We were specifically interested in the effects of sleep-onset on the subsequent ARSQ-ratings, therefore following each ECR trial, participants either underwent a sleep trial or stayed awake for the duration of a previous sleep trial (Figure 1). As with the resting-state trials, for each cognitive dimension we fitted a separate linear mixed model (LMM), in which its post-sleep or post-wake ARSQ-rating was regressed on EEG biomarkers and potential confounders, i.e., experimental day, experiment duration on a given day, and participant age. In addition to EEG biomarkers obtained from the wake and sleep trials and post-trial ARSQ-ratings, we included the EEG biomarkers and ARSQ-ratings from the preceding resting-state trial in these models, because significant correlations between dimensional scores over time were expected (Diaz et al., 2014). Analogous to the resting-state trials, the general form of these models, with subscript “i” denoting the corresponding ARSQ-dimension, was:

$$\begin{array}{l} {\text{ARSQ}}_{\text{i}}^{\text{Post-trial}}={\text{Trial}}_{\text{Sleep}}+{\text{EEG}}_{\text{within}}+{\text{EEG}}_{\text{between}}+\\ \;{\text{ARSQ}}_{\text{i, within}}^{\text{ECR}}+{\text{ARSQ}}_{\text{i},\text{ between}}^{\text{ECR}}+\\ \;\text{Age}+\text{Day}+\text{Duration}+\text{random effects} \end{array}$$

where “Trial_Sleep_” denotes an indicator variable designating whether or not the trial type was a sleep (1) or wake (0) trial.

The results (Table 2) of predicting post-trial cognition suggest that only on three ARSQ-dimensions did the transition from wakefulness to stage 1 sleep have a significant effect on cognition compared to the wake trials: ratings on Theory of Mind and Planning were both reduced, whereas Sleepiness—not surprisingly—was increased during sleep trials. The effects of the EEG biomarkers in each of these three models were limited to a significant between-subject effect of parietal theta power on post-sleep ratings of Sleepiness and a significant negative effect of frontal alpha-theta ratio on Theory of Mind. Finally, in line with our hypothesis, ratings of subjective experiences from the eyes-closed rest trial preceding the sleep trials on the other hand were highly significant predictors of subsequent ARSQ-ratings.

**Table 2:** **ARSQ-dimensions Theory of Mind, Planning, and Sleepiness are shown to be sensitive to sleep-onset**.

**Post-trial ARSQ- rating: (*n*_*sleep*_ = 144, *n*_*wake*_ = 79)**	**Theory of Mind**	**Planning**	**Sleepiness**
Degrees of Freedom^a^	15	17	17
**FIXED EFFECTS (COEF. EST**. ± **SE)**			
**Preceding ECR ARSQ rating**			
Within-subject effect	0.20 ± 0.06^***^	0.15 ± 0.05^**^	0.39 ± 0.05^***^
Between-subject effect	0.93 ± 0.28^***^	0.80 ± 0.18^***^	0.77 ± 0.09^***^
Sleep trial	−0.19 ± 0.09^*^	−0.34 ± 0.09^***^	0.27 ± 0.08^**^
**Alpha/Theta Ratio^b^**	**Frontal**		
Within-subject effect	−0.95 ± 0.43^*^		
Between-subject effect	−0.60 ± 0.34		
**Theta abs. power**			**Parietal**
Within-subject effect			0.22 ± 0.24
Between-subject effect			0.41 ± 0.16^*^
**RANDOM EFFECTS (SD)**			
Participant ID (β_0_)	0.32	0.26	0.10
Experimental day (β_1_)	0.49	0.15	0.26
Correlation β_0_,β_1_	−0.48	0.99	0.91
Residual	0.65	0.64	0.58
Explained variance ${\left(\Omega_{0}^{2}\right)}^{}$^c^	0.53	0.46	0.62

*NB, Shown are estimated model coefficients ± Standard Error (the fixed effects, excluding intercept term) and random effect estimates*.

a *Degrees of freedom and p-values based on Kenward-Roger approximation*.

b *EEG Biomarkers were within-group centered (van de Pol and Wright, 2009)*.

c *Approximates overall model fit, similar to R^2^ in classical regression (Xu, 2003)*.

* *p ≤ 0.05*,

** *p < 0.01*,

*** *p < 0.001*.

*Significant results in bold face*.

### Resting-state theta-activity and sleepiness ratings are associated with sleep-onset

The preceding analyses suggest that both resting-state ARSQ-ratings and in-trial EEG-measures, especially in the theta-band, may be used to predict post sleep-trial cognition. To assess whether the cognitive and electrophysiological data from the eyes-closed resting-state could also be used to predict the time to sleep onset, the so-called sleep onset latency (SOL), we specified a linear mixed model based on exclusively the sleep trials (*n*_sleep_ = 144) as follows:

$$\begin{array}{l} \text{SOL}={\text{EEG}}_{\text{within}}^{\text{ECR}}+{\text{EEG}}_{\text{between}}^{\text{ECR}}+{\text{ARSQ}}_{\text{within}}^{\text{ECR}}+{\text{ARSQ}}_{\text{between}}^{\text{ECR}}+\\ \;\text{Age}+\text{Day}+\text{Duration}+\text{random effects} \end{array}$$

where both EEG and ARSQ variables potentially include all relevant biomarkers and ARSQ-dimensions. However, after testing several models, only the post resting-state Sleepiness rating and the parietal theta-power DFA-exponent over the resting-state period appeared significant negative predictors of sleep-onset latency, predominantly through their within-subject components, apart from the included covariates. The effect of experimental day and the overall duration of the experimental session produced the strongest effect, confirming the profound habituation exhibited by participants (Table 3).

**Table 3:** **Sleep onset may be predicted from a combination of resting-state ARSQ-ratings on Sleepiness and the theta-band derived DFA-exponent**.

**(*n* = 144)**	**Sleep-onset latency [minutes]**
Degrees of freedom^a^	16
**FIXED EFFECTS (COEF. EST**. ± **SE)**	
Experiment day	−4.15 ± 1.36^**^
Experiment duration [hours]	−1.20 ± 0.15^***^
**Sleepiness**	
Within-subject effect	−0.60 ± 0.28^*^
Between-subject effect	−1.39 ± 0.95
**Theta DFA-exponent**^b^	**Parietal**
Within-subject effect	−13.53 ± 6.68^*^
Between-subject effect	36.24 ± 19.87
**RANDOM EFFECTS (*****SD*****)**	
Participant ID (β_0_)	4.0
Experimental day (β_1_)	4.68
Correlation β_0_,β_1_	−0.84
Residual	2.1
Explained variance ($\Omega_{0}^{2}$)^c^	0.83

*NB: Shown are estimated model coefficients ± Standard Error (the fixed effects, excluding intercept term) and random effect estimates*.

a *Degrees of freedom and p-values based on Kenward-Roger approximation*.

b *EEG Biomarkers were within-group centered (van de Pol and Wright, 2009)*.

c *Approximates overall model fit, similar to R^2^ in classical regression (Xu, 2003). ^c^Approximates overall model fit, similar to R^2^ in classical regression (Xu, 2003)*.

* *p ≤ 0.05*,

** *p < 0.01*,

*** *p < 0.001*.

*Significant results in bold face*.

## Discussion

Here, we have shown that alpha- and theta-band EEG measures are significant predictors of post resting-state ratings of Discontinuity of Mind (theta DFA-exponent and the ratio of alpha/theta power), Theory of Mind (alpha power), Self (theta power), Planning (alpha/theta ratio), Sleepiness (both theta power and alpha/theta ratio), and Comfort (alpha power). Although, previous studies have shown relationships between EEG power and spontaneous cognition using regular probes (Lehmann et al., 1995, 1998), to our knowledge, the current report is the first showing that variation in EEG can be linked to retroactive self-reports of subjective experiences in the classical eyes-closed rest condition.

Furthermore, the effects on ARSQ-ratings following the transitioning from wakefulness to S1 sleep were identified (Table 2). Although (severe) changes in conscious cognition are expected as individuals descend further into successive sleep stages (Rechtschaffen and Kales, 1968; Yang et al., 2010), our results show that at least during early stage 1 sleep, ARSQ ratings differ only on Theory of Mind and Planning (apart from Sleepiness itself) from the ratings obtained during wake trials. Although, an in-depth analysis of topographical dynamics was beyond the scope of the current study, the significant effects at frontal electrodes, especially the alpha/theta ratio, found for Theory of Mind may be in line with earlier reports suggesting frontal involvement for Theory of Mind related function (Sabbagh and Taylor, 2000; Shamay-Tsoory et al., 2005; Spreng et al., 2008; Spreng and Grady, 2009). In combination with the commonly observed frequency slowing and anterior-posterior shift in EEG-power during the transition toward sleep (Werth et al., 1997; De Gennaro et al., 2001), a disproportional decrease in theta-power at frontal electrodes could explain the observed increase in the alpha/theta and the associated decrease in ARSQ-ratings on Theory of Mind.

Interestingly, the expected effect of sleep-trial theta-power on Sleepiness was limited to differences between participants, due to very little variation in theta-power irrespective of the observed rating within an individual's observations. This is in contrast to the eyes-closed rest case, where parietal theta-power in relation to Sleepiness exhibited the opposite pattern of a dominant within-subject effect. These findings could suggest the existence of two different theta generating processes: one active during eyes-closed rest and individually associated with experienced sleepiness, whereas the other appears reminiscent of a default “background” process triggered during the sleep/wake trials, possibly due to the familiar context associated with sleeping (i.e., lying in bed for an extended duration), and—on an individual basis—not related to the experience of sleepiness at all. Interestingly, neither the duration of the trial nor the interaction (results not shown) between trial type (sleep or wake) and theta power were significant. A potential explanation may therefore lie in either the physical context differences between ECR and the sleep/wake trials (sitting versus supine position) or the subtle difference in instruction given: only during eyes-closed rest were participants instructed to avoid falling asleep.

By integrating the results from these two analyses a successful attempt was made to predict sleep-onset latency using only predictors derived from the preceding 5 min eyes-closed rest trial. Sleepiness proved a significant negative predictor, with each unit increase above the within-subject average lowering sleep-onset latency by roughly 40 s (see Table 3). Although, the effect of Sleepiness was anticipated, we would have expected that ARSQ-dimensions such as Discontinuity of Mind, Comfort, or Somatic Awareness to yield stronger effects. One possibility is that participants showed a proportional awareness of their own susceptibility to fall asleep, although the reliability of this self-awareness in terms of when participants would be likely to fall asleep, has been shown to be rather low (Kaplan et al., 2007)—which highlights the need to combine self-assessments with physiological markers. At the EEG level, the parietal theta DFA exponent was shown to be a particularly strong positive predictor of sleep-onset latency, with each unit increase above the within-group average leading to a potentially more than 13 min decrease in sleep-onset latency. Still, the raw magnitude of this effect should be interpreted with care, as full unit increases in the DFA-exponent are unlikely. It nevertheless does suggest that not only do increasing long-range temporal correlations in the parietal theta-band appear to be associated with faster transition toward sleep (with about 1.35 min per 0.1 increase in Theta DFA-exponent), but also that the DFA exponent could be a more sensitive measure of within-subject variability.

Despite, these results, certain caveats need to be considered, most prominently the effect of experimental day and duration of the experimental session. We observed strong habituation across trials with substantial variation both within and between individuals. Although, we specified our models to accommodate these effects by specifically allowing for individual trends across trials and correlation between random slopes and intercepts. For example, participants with relatively high initial sleep-onset latencies—resulting in higher global averages and intercept values—could rapidly habituate to much lower latencies, resulting in a steep negative trend correlated to the above average intercept. Another side effect of this sleep-trial habituation is that matching their duration for use in the wake trials becomes more of a challenge. This may in part explain the quite uniformly observed ratio of sleep to wake trials of ~1:2 (see Table S2). Future designs (see below) should take these effects into consideration to avoid potential overshadowing effects of these covariates. In addition, having access to participant sleep history and other chronotype related information may help refine methodology further. We do note however, that the arousal level of participants as assessed by alpha and theta power during the eyes-closed rest measurements did not differ with respect to the subsequent trial type, i.e., we did generally not observe for instance higher theta power during rest preceding a sleep trial (see Figure S4). Finally, the absence of reliable items probing visual and verbal experiences in the current study due to the use of the older ARSQ 1.0 unfortunately prevents relating our findings to the rich literature on hypnagogic experiences which appear to strongly feature visual mentation (Foulkes et al., 1966; Foulkes and Fleisher, 1975; Schacter, 1976; Hori et al., 1994; Tanaka et al., 1998; Wackermann et al., 2002). Analyses based on the single items “I thought in images” and “I thought in words,” respectively, could not be associated with sleep/wake trial EEG and effects were mostly limited to between-subject effects of preceding resting-state ratings (see Table S1).

The pioneering character and largely exploratory nature demanded certain methodological choices to be made. This primarily translates to the high degree of aggregation of the electrophysiological data. Reduction of the 256-channel EEG-recordings to a simple average of frontal, central, parietal and occipital 10–20 system regions, potentially foregoes much of the spatial dynamics associated with both the transition toward sleep as well as the resting-state. Similarly, the EEG biomarkers utilized here only focus on the total activity within a set period, discarding much of the temporal dynamics involved with changes in brain states (Lehmann et al., 1998). Although, this study focused on the alpha and theta band frequency ranges, it is important to note that gamma-band activity has been shown to play an important role in cognition (Fries, 2009; Köster et al., 2014; Roux and Uhlhaas, 2014) as well. More importantly, despite the already extensive reduction of EEG-channel data in the analyses, only single electrode locations were used in the statistical models instead of using all five locations. The reason behind this choice was that EEG biomarkers exhibited strong correlations (even at false-discovery rate corrected *p*-values at an α-value of 0.001) not only over channels but also between frequency bands (see Figure S3), which could lead to spurious or unstable effects potentially due to a high degree of collinearity. It should also be noted that given the observed effects, future studies may attempt to further dissect the EEG-activity of the transition period between wakefulness and S1 sleep, for instance by utilizing the subdivisions introduced by Hori (Hori et al., 1994; Tanaka et al., 1998). With regard to the participant sample, besides expanding the number of volunteers, including female participants would seem a logical step given the potentially interesting differences between sexes to be found (Van Der Sluis et al., 2010; Miller and Halpern, 2014). The motivation to exclusively employ male volunteers was primarily dictated by pragmatism: short hair significantly reduces preparation time and simplifies electrode adjustment.

These above caveats notwithstanding, the presented results suggest that resting-state cognition in combination with EEG may be used to predict sleep-onset latency. In order to confirm the practical utility of such a prediction, i.e., assess its reliability and accuracy, one may adapt the current experimental design by (1) expanding the participant pool while simultaneously decreasing the number of trials per participant and (2) abolish the matching between sleep and wake trials, giving all participants a fixed time interval to fall asleep (e.g., 30 min). This would open the possibility to use logistic regression to model the probability of falling asleep as a function of pre-trial resting-state cognition and EEG biomarkers. Provided a useful relationship can be established, such a result could then eventually be translated into working prototypes of neurofeedback equipment aimed at facilitating sleep-onset. For instance, a device that probes an individual's sleepiness in combination with processing theta-band activity may be able to provide a forecast of how long it would take to fall asleep—potentially quite useful source of information for the numerous individuals suffering from sleep-onset insomnia (Ohayon, 2002; Van der Heijden et al., 2007). The device could, for example, recommend alternative activities to going to bed if the person is not in a suitable combination of EEG and cognitive state for a short sleep-onset latency. While the efficacy of neurofeedback focused on regulation of inner states, for example in the treatment of ADHD-symptoms (Lofthouse et al., 2011), has been less self-evident (Diaz et al., 2012), neurofeedback in the form of brain-computer interfaces has booked tremendous successes in the past decade by harnessing reliable physiological signals such as P300 evoked potentials and slow-cortical potentials providing relief for certain groups of patients unable to otherwise communicate with their environment (Birbaumer et al., 1999; Kleih et al., 2011). The results presented here may be viewed as one possible route toward the development of a health-monitoring device for individuals suffering from sleep-onset insomnia, possibly in conjunction with existing therapies, providing a useable estimate of the likelihood to successfully fall asleep in combination with advice on what to do to promote sleep onset.

## Author contributions

AD: experimental design, data collection, data analysis, writing. RH: data analysis, writing. HDM: writing, laboratory facilities, expert counsel. EVS: experimental design, writing, laboratory equipment, expert counsel. KLH: research project lead, writing.

### Conflict of interest statement

The authors declare that the research was conducted in the absence of any commercial or financial relationships that could be construed as a potential conflict of interest.

## Footnotes

^1^Prior to analyzing the data with linear-mixed models (LMM) we compared the LMM fit to the same fit using classical linear models without random term, to test the possibility of intra-individual variability being on par with inter-individual variability which would suggest that observations could be treated as independent. In all cases, linear mixed models provided a significantly better fit.
