# Supplementary material for: Resting-State Subjective Experience and EEG Biomarkers Are Associated with Sleep-Onset Latency
Source: Front Psychol. 2016 Apr 12;7:492. doi: 10.3389/fpsyg.2016.00492 (PMC4828461; doi:10.3389/fpsyg.2016.00492)
Supplement: Supplementary file 1 [file DataSheet1.PDF]

Supplementary Material

Resting-state subjective experience and EEG biomarkers are associated with sleep-onset latency

B. Alexander Diaz[a,d], Richard Hardstone[a,d], Huibert D. Mansvelder[a,d], Eus J.W. Van Someren[a,b,c,d], and Klaus Linkenkaer-Hansen[a,d*]

[a]Department of Integrative Neurophysiology, Center for Neurogenomics and Cognitive Research (CNCR), Vrije Universiteit Amsterdam, De Boelelaan 1085, 1081 HV Amsterdam, The Netherlands.
[b]Department of Sleep & Cognition, Netherlands Institute for Neuroscience, Meibergdreef 47, 1105 BA Amsterdam, The Netherlands.
[c]Department of Medical Psychology, VU University Medical Center, 1Y 156, De Boelelaan 1117, 1081 HZ Amsterdam
[d]Neuroscience Campus Amsterdam (NCA), De Boelelaan 1085, 1081 HV Amsterdam, The Netherlands.

Table S1. The single ARSQ 1.0 items addressing subjective experience with respect to visual or verbal thoughts post sleep/wake trial are almost exclusively associated with preceding resting-state ratings. In addition, imagery appeared to increase with experimental duration.

Post-ECR ARSQ-rating: (*n* = 223)	**"I though in images"**	**"I thought in words"**
Degrees of Freedom [1)]	35	8
Fixed effects (est. ± SE)		
Pre-trial ARSQ rating		
Within-subject effect	**.00±.06**	**.08±.06**
Between-subject effect	**.70±.10*** **	**1.03±.08*** **
Duration [hrs.]	**.09±.03** **	
Random effects (SD)		
Participant ID (β_0)	.30	.06
Day (β_1)	.52	.27
Correlation β_0,β_1	*-.72*	*1.0*
Residual	.69	.79
Explained variance (Ω_0^2) [3)]	.51	.60

[1)] Degrees of freedom and p-values based on Kenward-Roger approximation.[2)] EEG Biomarkers were within-group centered (van de Pol and Wright, 2009).[3)] Approximates overall model fit, similar to R^2 in classical regression *(Xu, 2003). Abbreviations: * p ≤ .05, **p < .01, *** p < .001*

Table S2. Although the proportion of observed sleep to wake trials is approximately 2:1 (144:79), this distribution does not seem to be caused by (severe) distortions from specific participants. Even across experimental days, a χ^2 -test for equal proportions did not reveal a significant difference (χ^2(1,3.57) , p = 0.059).

Participant	Total trials	Total sleep trials	Proportion sleep trials
1	18	11	0.61
2	18	9	0.50
3	18	11	0.61
4	18	12	0.67
5	16	10	0.63
6	18	11	0.61
7	18	12	0.67
8	18	12	0.67
9	18	12	0.67
10	9	6	0.67
11	18	15	0.83
12	18	11	0.61
13	18	12	0.67

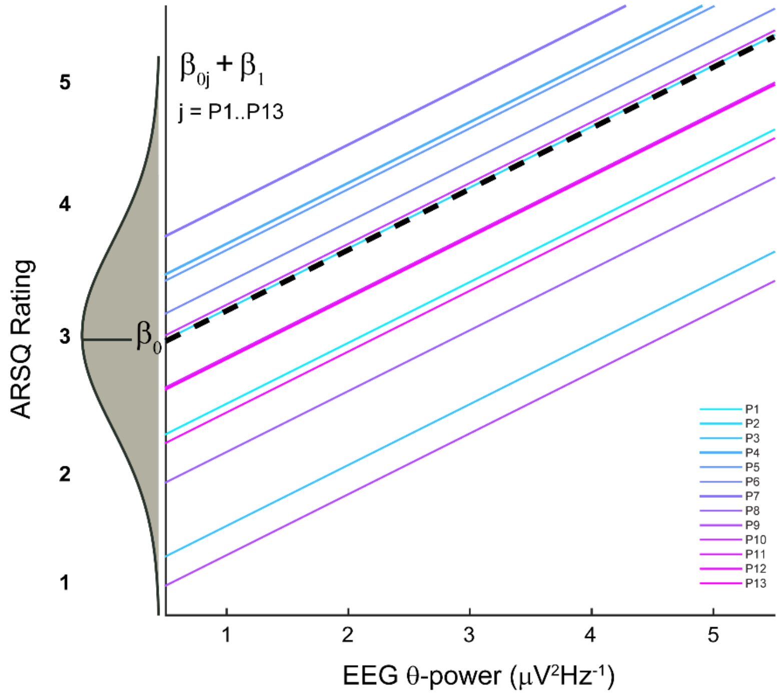

Figure S1 Hypothetical example of random *intercept* variation among individuals P1-P13 with repeated observations. Each individual has its own collection of observations (not shown) to which a least squares regression line is fit (colored lines). Within the linear mixed model framework, individuals may exhibit random normal variation in the intercept (β_{0i}) only (assuming a fixed slope, i.e. $\beta_{1i} = 0$), with respect to an overall mean (dashed line). Alternatively, one may modify this model by allowing variation in the slope ($\beta_{1i} \neq 0$) as well (i.e., non-parallel regression lines). Treating individual variation as random effect provides such benefits as generalization to larger populations and tolerance against missing observations within individuals. Especially the latter condition would lead to complete rejection of a subject's data within classical regression or ANOVA frameworks.

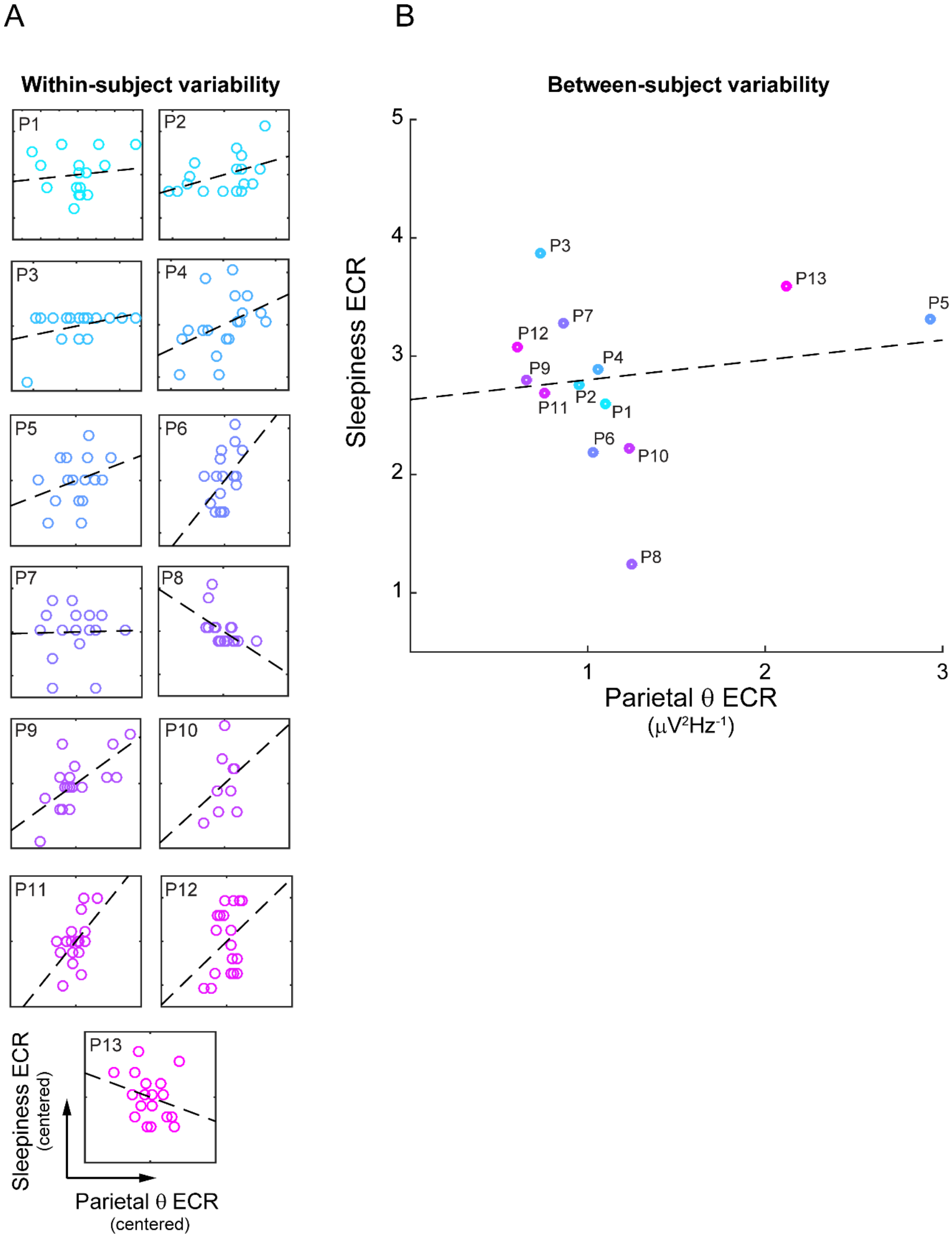

Figure S2 Illustration of within-subject and between-subject variability in the relationship between total absolute theta power at parietal locations obtained during 5 minutes eyes-closed rest (ECR) and the immediately following ARSQ rating on Sleepiness. Within-group centering (van de Pol and Wright, 2009) reveals a mostly significant positive association between theta power and Sleepiness score (***A***), whereas in (***B***) this effect across participants is much more attenuated (P1-13) as represented by their group averages (i.e., observations within a participant).

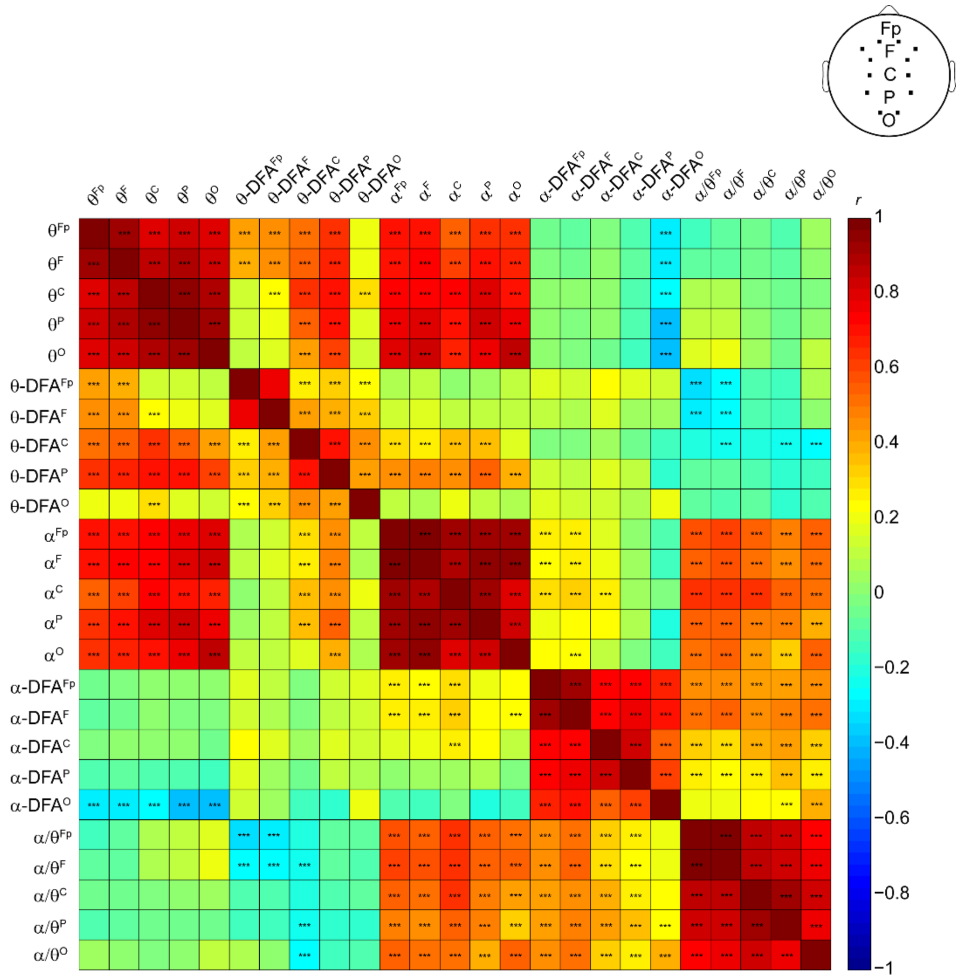

n = 223

*** *p* <.001 (FDR-corrected)

Figure S3 Strong correlations among and between EEG biomarkers were observed during eyes-closed rest recordings (pooled data set), potentially degrading statistical models due to collinearity. As countermeasure, only a single electrode location for a given biomarker was used in each model.

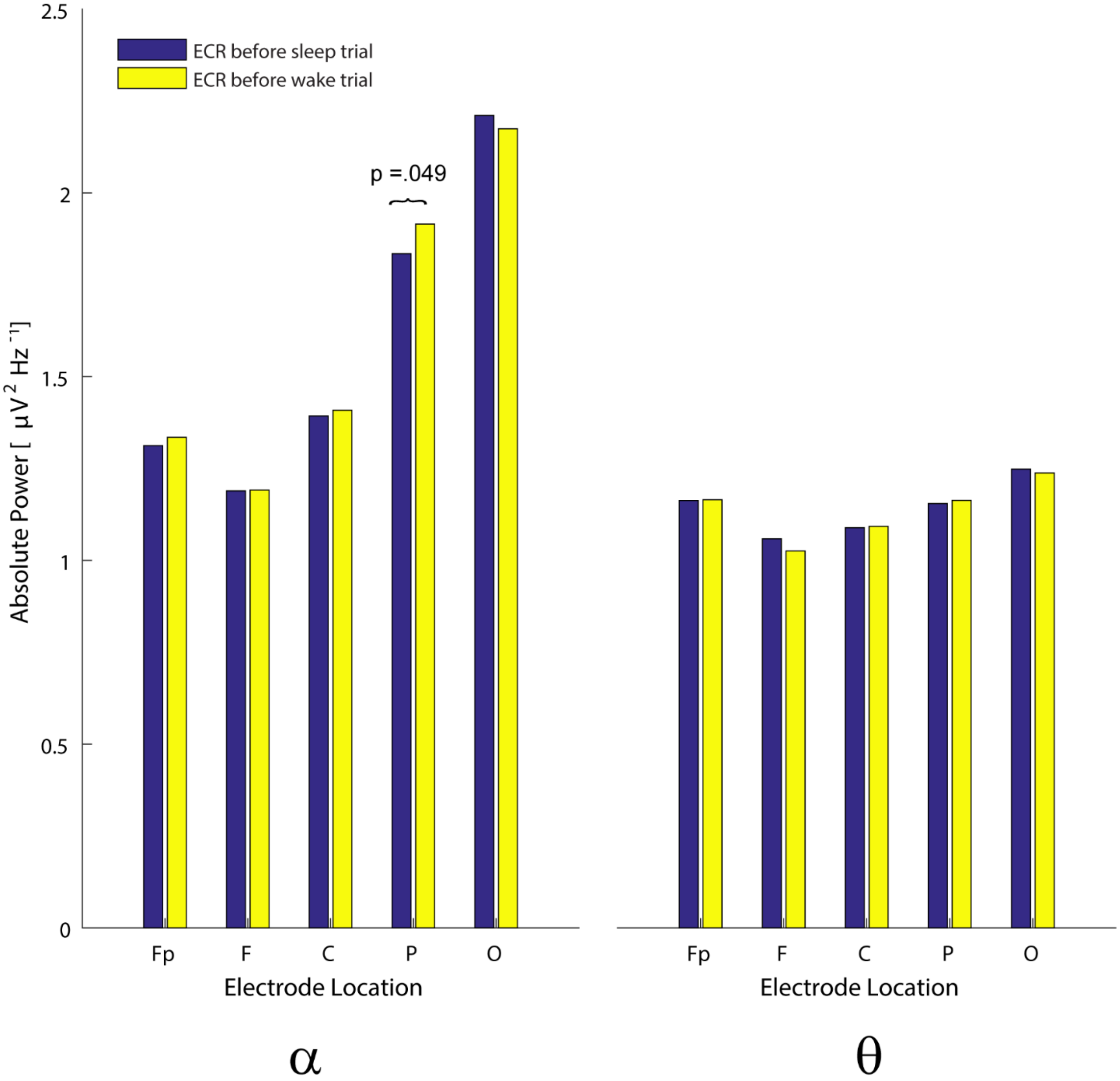

Figure S4 Absolute band-power in the alpha and theta range during 5 minutes eyes-closed rest is generally independent of the subsequent trial type, i.e. sleep or wake.
